# Views of Old Age and Death Held by Working-age Men and Women and Their Relationship to Health-related Behavior

**DOI:** 10.2188/jea.14.23

**Published:** 2005-03-18

**Authors:** Atsushi Hioki, Tagayasu Tanaka

**Affiliations:** 1 Gifu Prefectural Gifu Region Public Health Center.; 2 Gifu Prefectural Institute of Health and Environmental Sciences.

**Keywords:** death, old age, life expectancy, personal satisfaction, health behavior

## Abstract

BACKGROUND: Many working-age people have poor health habits. The aim of this study was to investigate their views of old age and death and the relationship of those views to their health habits.

METHODS: A structured interview about views on old age and death, self-rated health, satisfaction with life (life satisfaction) and health habits was conducted on a random sample of 1,200 men and women aged 30-59 years in the southern part of Gifu Prefecture, Japan. The response rate was 78%.

RESULTS: Less than half as many respondents expected to live past 80 years than expected to live past 70 years. A greater percentage of men than women had no plan for old age, while the percentage of women who expected to live with friends and family was higher than that for men. For men, fewer symptoms, life satisfaction, valuing health, and anxiety about health status during solitary old age, and for women, occupation, fewer symptoms, life satisfaction, valuing health, expecting social participation during old age, expecting to live with her spouse during old age and considering one’s own death were positively associated with health habits. Experience of presence at a deathbed was not related to health habits.

CONCLUSIONS: Results of our survey suggest that men have a poor outlook toward old age and death and suggest the possibility that helping men prepare for an inevitable death may help them live fuller lives.

Although death naturally and inevitably ends the human experience, young people in Japan are less often witnessing the last moment of a grandfather, grandmother, or other person because of the increase in the number of nuclear families^1^ and decrease in the percentage of people that choose to die at home. The percentage showed a dramatic decrease from 1960 through 1980, 70.7% to 38.0% in Japan; that is, nearly half as many people chose to die at home. From 1980 through 2000, the percentage dropped to 13.9%.^2^ In addition, the Japanese people are not very religious,^3^^,^^4^ although they conduct funerals according to Buddhist traditions, marriage ceremonies according to Shinto traditions, pay their respects to a Shinto shrine at the beginning of the year, and cerebrate Christmas at the end of the year. It has been pointed out that many young Japanese are not educated about the meanings of life and death through religious training or at school and that they are not interested in death.^5^

Concern over one’s death also implies a concern over preparations for aging. A good death can be achieved via maintaining good health and being satisfied with life.^6^^,^^7^ It has been proposed that people prepare themselves for death just as they prepare themselves for life. Education in preparation for death, however, has been limited to end-of-life care.

Many working-age people who are busy with work and raising children and who consider themselves healthy have poor health habits.^8^ We hypothesized that having been present at a deathbed or a person’s views of old-age and death influence preparations for aging and health habits, and that the views held by many working-age people have undesirable consequences when they reach old age. In addition, a cohort study^9^ noted that those with more a positive perception of themselves as they age live longer when age, gender, socioeconomic status, loneliness and functional health are controlled for, partially mediated by the will to live. Because there has been no report of the relationship between health habits and feelings about old age and death, we undertook this study to determine whether such a relationship exists. Also, we sought to identify measures likely to be useful in encouraging good health habits.

## METHODS

The study area is the Gifu Region, located in the south of Gifu Prefecture, Japan, and in 2000 included 17 municipalities: 3 cities, 12 towns, and 2 villages. The area of the region is 992 km^2^ and the population was 795,000.^10^ The percentage of the population aged 65 years and older was 15.9%.

We conducted a cross-sectional survey using a structured interview. Respondents expressed their views toward old age and death, self-rated their health and quality of life, and self-reported symptoms and health habits.

We randomly selected 1,200 men and women aged 30-59 years living in the region by a two-stage sampling procedure. The first step was to select 15 municipal office and branch office areas from 23 areas where the Basic Resident Registers were kept (office areas). The second step was to choose 40-200 individuals randomly from the Basic Resident Registers of each of the 15 areas in proportion to the population. We mailed a request for a personal interview to each subject. Trained female interviewers then conducted the interviews in February, 2002, and 926 of the 1200 (78%) men and women contacted responded. The questionnaires were completed so that the subjects remained anonymous.

### Questionnaire

Guided by the questionnaire, subjects self-rated their health, self-reported their symptoms, and reported their degree of satisfaction with life (life satisfaction), sense of contributing to the life of another person, health habits, experience of presence at a deathbed, and views on old age and death. As background information, we gathered data on age, occupational status, person with whom the subject lived (household member), and whether the subject lived in his/her own home.

Respondents self-rated their health by selecting one of four options (very poor, somewhat poor, good, and very good). From a list of symptoms (shoulder pain, back pain, joint pain, constipation/diarrhea, numbness, headache, stomach pain, palpitation/shortness of breath, fatigue, forgetfulness, being quick tempered, dizziness, sensitive to cold, hypertension/hypotension, and eye strain), they selected one of three options (without, sometimes, and always) to characterize the frequency of each of these symptoms.

They self-rated the degree to which they were satisfied with their health status, health habits, employment, participation in social activities, leisure activities, friendship, support from neighbors, and economic status by selecting one of five options (very dissatisfied, somewhat dissatisfied, intermediate, satisfied, and very satisfied) for each of the 8 categories. We counted the items for which the response was “satisfied” or “very satisfied” as “satisfied” to create a life-satisfaction score (minimum 0, maximum 8). Respondents expressed how often they had a feeling of contributing to another person’s life by selecting from among five options (never, scarcely ever, occasionally, often, and always). They assessed their health habits by answering “true” or “false” to 8 statements: I do not smoke; I do not consume alcohol excessively; I eat breakfast every morning; I sleep for 7 to 8 hours; I work for less than 9 hours each day; I get physical exercise twice a week or more; I eat a balanced diet; and I have little mental stress.^11^^,^^12^ We counted the items for which the response was “true,” and designated it the health-practice score (minimum 0; maximum 8).

To characterize the views of our subjects toward old age and death, we asked the following questions (suggested responses are listed in parentheses):

(1) How long do you expect to live? (less than 70 years, 70-79 years, 80-89 years, 90 years or more, unknown)
(2) During old age, which of the following activities do you expect will best characterize your daily life? (hobby, participation in social activities, friendship, work, no plan)
(3) During old age, whom do you expect to live with? (spouse, children and/or grandchildren, friends)
(4) What makes you anxious about being alone during old age? (cooking, cleaning and washing, health status, economics, traffic situation, friendship, not anxious)
(5) How do you feel about your own inevitable death? (fearless, fearless though anxious, fearful, trying to accept, never considered)
(6) What will happen to you after you die? (back to nature, nothing remains, heaven or hell, exist in the presence of God, my soul will transmigrate, never considered)

### Statistical Analysis

Age differences were assessed with the Chi-square test and differences between men and women with the Mantel-Haenszel procedure. The health-practice score, the number of self-reported symptoms, and the life-satisfaction score were compared by analysis of variance (ANOVA). We used multiple regression analysis to assess the influence on health habits of the views held toward old age and death. The independent variable was the health-practice score. As dependent variables, we selected significant items by preliminary multiple regression analysis from age, occupation, household member, living in one’s own house, self-rated health (1-4 with increasing values indicating an increase in self-rated health), number of self-reported symptoms, life-satisfaction score, the most often selected item of the 8 life-satisfaction categories, and feeling of contributing to the life of another (from “never” = 1 to “always” = 5). Dummy variables were used for occupation, taking “employee or public official” as the reference. We added the variables that related significantly to the health-practice score by ANOVA from the experience of presence at a deathbed, self-estimated life expectancy, expected daily lifestyle during old age, person expected to live with during old age, sense of one’s own death, and one’s conception of the afterlife; then we re-analyzed the variables. Dummy variables were used for “sense of one’s own death” taking “never considered” as the reference.

## RESULTS

Table 1 shows results of the questionnaire for health habits, self-rated health, self-reported symptoms and life satisfaction. The mean health-practice score was higher for women (5.50) than for men (4.64). Among men, those in their fifties had the highest score (p<0.01). Among women, the percentage of good self-rated health declined for each successively older age group; this was also true for men but without statistical significance. The number of self-reported symptoms (always or sometimes) was higher for women than for men (p<0.01). There was no significant age difference in the number of self-reported symptoms according to age in either men or women, although the value increased with each age category in men. Among the self-reported symptoms, back pain (26%), shoulder pain (25%), and eye strain (23%) in men, and shoulder pain (37%), eye strain (21%) and fatigue (21%) in women were reported in more than 20% of the respondents. The mean life-satisfaction score was higher for women (4.04) than for men (3.35) (p<0.01). The fraction of women who regarded health as most important was higher than that of men and increased with age in both men and women (p<0.01).

**Table 1.** Summary of health practices, self-rated health, self-reported symptoms, life satisfaction, and feeling of contributing to life of another of randomly selected working-age persons, by sex and age.

	All subjects		Men							Women							p (sex) ^†^
	
			30-39 y.o.		40-49 y.o.		50-59 y.o.		p (age) *	30-39 y.o.		40-49 y.o.		50-59 y.o.		p (age) *	
n	926		133		143		160			161		154		175			
Mean of health-practice score ^‡^	5.09	(1.45)	4.52	(1.43)	4.43	(1.49)	4.93	(1.44)	0.006	5.59	(1.19)	5.29	(1.44)	5.59	(1.29)	0.067	0.000
Good self-rated health (%)	88.8		90.2		88.1		85.0		0.391	93.8		91.6		84.6		0.014	0.369
Mean of self-reported symptoms ^§^	1.87	(2.01)	1.38	(1.79)	1.69	(1.58)	1.83	(1.93)	0.09	1.80	(1.94)	2.30	(2.21)	2.09	(2.32)	0.125	0.002
Mean of life-satisfaction score ^||^	3.72	(2.39)	3.09	(2.42)	3.05	(2.34)	3.83	(2.42)	0.006	4.06	(2.29)	3.74	(2.24)	4.29	(2.35)	0.096	0.000
Regarding health as most important (%)	60.5		42.1		55.2		63.1		0.001	57.8		63.0		76.6		0.001	0.000
Always or often feeling as if contributing to life of another (%)	30.9		26.3		30.1		35.0		0.27	28.0		34.4		30.9		0.463	0.949

* P-values by contingency table for percentage between age groups or by analysis of variance for mean value.

^†^ P-values by Mantel-Haenszel procedure for percentage between men and women or by unpaired t-test for mean value.

^‡^ Total number (0-8) of corresponding items (not smoking; not consuming alcohol excessively; eating breakfast; sleeping for 7 to 8 hours per day; working for less than 9 hours; regular physical exercise; nutritional balance; and little mental stress).

^§^ Total number (0-15) of 15 symptoms (having always or often).

^||^ Sum (0-8) of 8 items (health status; self-rated health; employment; participating in social activities; leisure activities; friendship; support by neighbors; and economic status).

Standard deviations in parentheses.

Table 2 shows the experience of presence at a deathbed and views toward old age and death held by the respondents. Sixty-one percent of respondents had been present at the last moment of another person; percentages were significantly higher for women than for men (p<0.05). We also found that the older the subjects, the higher was this percentage (p<0.01).

**Table 2.** Experience of presence at a deathbed and views on old age and death held by a random sample of working-age persons, by sex and age. (%)

	All subjects	Men				Women				
	
		30-39 y.o.	40-49 y.o.	50-59 y.o.	p (age) *	30-39 y.o.	40-49 y.o.	50-59 y.o.	p (age) *	p (sex) ^†^
Experience of presence at a deathbed	61.1	39.1	62.2	68.8	0.000	49.1	67.5	75.4	0.000	0.024
Expect to live past 80 years	24.3	16.5	24.5	20.6	0.266	31.1	24.7	26.9	0.434	0.018
Expected lifestyle during old age ^‡^										
Hobby	42.4	46.6	46.9	43.1	0.765	41.6	44.8	33.7	0.103	0.095
Social participation	14.8	7.5	13.3	20.0	0.009	9.9	18.8	17.7	0.056	0.519
Friendship	54.8	39.1	39.2	44.4	0.562	67.1	66.9	66.9	0.999	0.000
Work	10.3	8.3	8.4	16.3	0.041	8.7	7.8	11.4	0.495	0.425
No plan	13.9	21.1	18.2	14.4	0.322	14.9	9.7	7.4	0.077	0.002
Expected person to live with during old age ^‡^										
Spouse	77.2	80.5	83.9	84.4	0.634	73.9	76.6	66.3	0.092	0.000
Children and/or grandchildren	56.4	48.1	50.3	49.4	0.934	62.1	63.0	62.9	0.985	0.000
Friends	23.7	18.0	13.3	10.6	0.182	36.0	39.0	23.4	0.005	0.000
Cause of anxiety about being alone during old age ^‡^										
Cooking	23.1	36.1	35.0	36.9	0.942	9.9	11.7	13.1	0.658	0.000
Cleaning and washing	13.5	21.8	21.0	23.1	0.902	7.5	6.5	4.0	0.383	0.000
Health status	65.7	53.4	64.3	65.6	0.069	62.7	71.4	73.7	0.074	0.011
Economics	42.9	35.3	42.7	25.6	0.007	60.2	58.4	34.9	0.000	0.000
Traffic situation	5.8	4.5	5.6	3.8	0.745	4.3	7.8	8.6	0.277	0.157
Friendship	30.3	25.6	26.6	28.1	0.882	33.5	38.3	29.1	0.213	0.033
Not anxious	5.2	9.0	2.8	7.5	0.085	3.7	5.2	3.4	0.694	0.139
Sense of one’s own death										
Fearless	10.9	9.8	12.6	15.6	0.327	6.8	11.0	9.7	0.414	0.102
Fearless though anxious	21.7	20.3	20.3	23.1	0.785	16.8	24.7	24.6	0.144	0.823
Fearful	11.7	12.0	11.2	10.6	0.930	11.8	13.6	10.9	0.737	0.783
Trying to accept	25.7	20.3	24.5	21.9	0.700	26.1	29.2	30.9	0.621	0.026
Never considered	30.0	37.6	31.5	28.8	0.263	38.5	21.4	24.0	0.001	0.139
Notion about the afterlife										
Go back to nature	33.0	27.8	33.6	36.9	0.257	24.8	35.7	38.3	0.023	0.988
Nothing remains	16.6	19.5	22.4	21.3	0.846	14.3	9.1	14.3	0.275	0.001
Heaven or hell	3.9	6.8	2.8	1.9	0.068	5.6	3.9	2.9	0.445	0.924
Exist with God	6.8	4.5	5.6	3.8	0.745	11.2	9.1	6.3	0.281	0.018
Transmigration of the soul	16.8	17.3	14.7	11.9	0.420	27.3	19.5	10.9	0.001	0.092
Never considered	22.4	22.6	19.6	24.4	0.602	16.8	22.7	27.4	0.065	0.978

* P-values by contingency table for percentage between age groups.

^†^ P-values by Mantel-Haenszel procedure for percentage between men and women.

^‡^ Multiple answers.

As to self-estimates of life expectancy, 21% of men and 24% of women answered “unknown.” Among men who estimated their life expectancy, 80% of respondents expected to live past 70 years, whereas only 26% expected to live past 80 years. For women, 88% of respondents expected to live past 70 years, whereas 36% expected to live past 80 years. These percentages were higher for women than for men (p<0.01). Percentages of estimated life expectancy did not differ significantly according to the age categories of either men or women. Concerning the expected lifestyle in one’s old age, a higher percentage of women selected “friendship” than did men (p<0.01), while a higher percentage of men selected “no plan” (p<0.01). The percentages of men expecting “to participate in social activities” and “work” were highest in the group aged 50-59 years. The percentage of men expecting to live with a spouse in old age was higher than that of women (p<0.01), but the percentage of women expecting to live with children, grandchildren or friends was higher than that of men (p<0.01). Responses to this item did not differ according to age in men, but the percentage of women expecting to live with friends was lowest in their fifties. Concerning what made them anxious about being alone during old age, men were significantly more likely than women to be anxious about cooking, cleaning and washing (p<0.01). Conversely, women were more likely than men to be anxious about health status, economics, and friendship (p<0.05). Both men and women in their fifties were less anxious about economics. There were no age differences with regard to other anxieties.

As to a sense of one’s own death, the percentage of women who were trying to accept their own death was higher than that of men (p<0.05). The percentage of women in their thirties who had never considered their own death was significantly higher in comparison with women in the older age groups (p<0.01). Concerning the afterlife, a higher percentage of men replied “nothing remained” than women (p<0.01).

There was no significant difference in the mean health-practice scores between men and women who had and had not been present at a deathbed (Table 3).

**Table 3.** Mean (standard deviation) of health-practice score by the views on old age and death.

	Men	Women
Total	4.64 (1.46)	5.50 (1.31)
Experience of presence at a deathbed	4.61 (1.50)	5.49 (1.38)
Expect to live past 80 years	4.97 (1.46)*	5.73 (1.23) *
Expected lifestyle during old age ^†^		
Hobby	4.64 (1.47)	5.55 (1.26)
Social participation	5.13 (1.59)**	5.41 (1.43)
Friendship	4.75 (1.42)	5.65 (1.25) ***
Work	4.74 (1.50)	5.15 (1.30)
No plan	4.35 (1.36)	5.06 (1.32) *
Expected person to live with during old age ^†^		
Spouse	4.67 (1.48)	5.64 (1.29) ***
Children and/or grandchildren	4.82 (1.37) *	5.48 (1.30)
Friends	4.83 (1.55)	5.50 (1.33)
Cause of anxiety about being alone during old age ^†^		
Cooking	4.69 (1.51)	5.56 (1.28)
Cleaning and washing	4.62 (1.45)	5.62 (1.03)
Health status	4.79 (1.36) **	5.53 (1.29)
Economics	4.42 (1.54) *	5.38 (1.33) *
Traffic situation	4.90 (1.79)	5.91 (1.12)
Friendship	4.61 (1.30)	5.49 (1.31)
Not anxious	4.46 (1.84)	5.45 (1.47)
Sense of one’s own death		
Fearless	4.73 (1.47)	5.27 (1.42)
Fearless though anxious	4.44 (1.58)	5.53 (1.37)
Fearful	4.20 (1.25) *	5.32 (1.50)
Trying to accept	4.74 (1.52)	5.81 (1.22) ***
Never considered	4.82 (1.37)	5.30 (1.16) *
Notion about the afterlife		
Go back to nature	4.83 (1.43)	5.64 (1.35)
Nothing remains	4.59 (1.52)	5.45 (1.25)
Heaven or hell	4.47 (1.42)	5.30 (1.23)
Exist with God	4.20 (1.47)	5.61 (1.43)
Transmigration of the soul	4.49 (1.55)	5.50 (1.26)
Never considered	4.61 (1.32)	5.30 (1.27)

* P<0.05, **P<0.01, ***P<0.001 vs. others by unpaired t-test.

^†^Multiple answers.

Both men and women who expected to live past 80 years had better health habits than those who did not (p<0.05). As to daily life during old age, men who expected to participate in social activities and women who expected friendship had better health habits (p<0.01). The mean health-practice score was higher for men who anticipated living with their children and/or grandchildren during old age and for women who anticipated living with their spouse. Men who were anxious about their health status during solitary old age had better health habits, although both men and women who were anxious about economics had worse health habits.

Concerning a sense of one’s own death, men who feared death and women who had never considered death reported worse health habits. In contrast, women who were trying to accept death had better health habits. The notion of an afterlife had no significant affect on the mean health practice score.

Table 4 shows relationships of lifestyle, health status, life satisfaction, and the views held toward old age and death to the health-practice score. For men, health-practice scores were positively correlated with three variables (life satisfaction, regarding health as most important, and anxiety about health status during solitary old age) and negatively correlated with two variables (self-reported symptoms and fear for one’s own death). For women, health-practice scores were positively correlated with six variables (occupation, life satisfaction, regarding health as most important, expecting to participate in social activities during old age, expecting to live with spouse during old age, and a sense of one’s own death) and negatively correlated with self-reported symptoms.

**Table 4.** Relationship of views on old age and death to health-practice score.

	Men				Women			
	
	Correlation coefficient		Standardized regression coefficient		Correlation coefficient		Standardized regression coefficient	
Occupation								
Employee or public official	-0.03		NU		-0.13	**	reference	
Self-employed	0.03		NU		-0.12	**	-0.02	
Part-time worker	0.02		NU		0.03		0.13	*
Housewife	-		NU		0.17	***	0.2	***
Without occupation	-0.02		NU		0.00		0.08	
Living in one’s own house	0.11	*	0.08		0.08		NU	
Number of self-reported symptoms	-0.21	***	-0.12	*	-0.28	**	-0.21	***
Life-satisfaction score	0.31	***	0.22	***	0.33	***	0.20	***
Regarding health as most important	0.20	***	0.16	***	0.15	***	0.10	*
Expect to live past 80 years	0.11	*	0.04		0.11	*	0.05	
Expected lifestyle during old age								
Social participation	0.13	**	0.09		-0.03		NU	
Friendship	0.06		NU		0.16	***	0.09	*
No plan	0.05		NU		-0.12	*	NU	
Expected person to live with during old age								
Spouse	0.04		NU		0.17	***	0.13	**
Children and/or grandchildren	0.12	*	0.05		-0.02		NU	
Cause of anxiety about being alone during old age								
Health status	0.13	**	0.10	*	0.04		NU	
Economics	-0.11	*	-0.06		-0.09	*	-0.06	
Sense of one’s own death								
Fearless	0.02		-0.05		-0.06		0.02	
Fearless though anxious	-0.07		-0.08		0.01		0.11	*
Fearful	-0.11	*	-0.10	*	-0.05		0.03	
Trying to accept	0.04		-0.03		0.15	***	0.14	**
Never considered	0.09		reference		-0.09	*	reference	

Multiple correlation coefficient	-		0.44	***	-		0.50	***

NU: not used for multiple regression analysis.

* P<0.05, **P<0.01, ***P<0.001.

## DISCUSSION

According to the life-table analysis of the Japanese population for 2000,^13^ the life expectancy at birth was announced to be 77.7 years and 84.6 years for men and women, respectively. In our survey, less than half as many respondents expected to live past 80 years than expected to live past 70 years. This indicates an underestimation of the likelihood of living past 80 years. There were no differences in expected life span according to the age of respondents. It was reported that the percentage of those who need support in daily life increased with advancing age, particularly in those aged 80 years or more.^14^ Our present results suggest the possibility that people are not anxious about support that may be needed in old age despite evidence that such support will be needed.

A greater percentage of men than women had no plan for their old age although the percentage decreased with advancing age in both sexes. More women than men indicated that they expected friendship as part of their lifestyle in old age. Men expected to depend on their spouses during the years following retirement whereas women expected to depend also on their children, grandchildren, and/or friends. Our survey results suggest the possibility that men do not adequately plan for their old age and death. Many men would be distraught and become severely depressed if their wives would die suddenly, leaving them alone, and subsequently their health would suffer.^15^^-^^17^ People also should prepare themselves for the eventual death of their spouses, close relatives and dear friends in order to prevent deterioration of their own health resulting from the death of dear persons. Men were anxious about their circumstances of daily life such as cooking, cleaning and washing. On the other hand, women appeared to be more likely to confront and deal with the anxiety they felt about health status and friendship. The percentage of women who were anxious about their economic status during old age was lowest in their fifties. Whether this is due to differences in the generations or changes that take place during aging cannot be determined from our survey.

The changes in views toward old age and death of the respondents by age were not prominent, although the percentage of those who had been present at a deathbed increased considerably with age in both men and women. The percentage of those without a plan for old age, however, tended to decrease with age, although not with significance.

The present study was cross-sectional, and the results do not explain the influence of views of old age and death on a long and healthy life. However, this survey was conducted by random sampling, and the response rate was relatively high. Although the ratio of the self-employed in the respondents was slightly lower (about 9% for men, and 6% for women) than that in the 2000 Population Census for those aged 30-59 years, the ratios of married subjects were similar.^10^^,^^18^ It is therefore likely that the respondents are representative of the total population of the region.

We evaluated the views of old age and death and analyzed the relationship of the views to present health habits. Health habits of men were poorer than those of women in our study population, supporting what was already reported.^19^^,^^20^ The percentage of those regarding health as most important was higher in women than in men. In this survey, the following were positively associated with good health practices; fewer self-reported symptoms, life satisfaction, and regarding health as most important in both men and women, anxiety about health status during solitary old age in men, and occupation, expecting to live with spouse during old age, and sense of one’s own death in women. It has already been reported that sociodemographic characteristics such as gender, age, education, marital status, health status and social network are associated with health practices.^19^^-^^21^

We hypothesized that being present at the last moment of another might induce one to consider one’s own death and therefore to live the remaining years more fully. However, such an experience showed no association with health habits. We did not ask whose last moment the respondent attended. The relationship with the deceased may determine whether presence at death provides an opportunity to consider one’s own desirable death or is a risk factor for poor health due to bereavement. The experience did not influence the fear of dying.

In this study, we showed that views toward old age and death were associated with health habits. Anxiety over health status during solitary old age was correlated positively in men, and expecting social participation during old age and expecting to live with one’s spouse during old age were correlated positively with health habits in women. These results indicate that views on old age may influence health habits. Fear of one’s own death was negatively correlated with health habits in men. In contrast, trying to accept one’s own death and being fearless though anxious about one’s own death were positively correlated with health habits in women. These results suggest the possibility that having an adequate conception of death and eliminating the fear of death via a proper understanding of death positively influence the likelihood of practicing a good health habits.

We can help people to plan for their old age, including their death,^22^ and to take control of their health. Self-rated health has been found to be a predictor of morbidity and mortality even after controlling for present disease or dysfunction.^23^^-^^26^ Mental and spiritual health status also have been reported to be associated with better health outcomes.^27^^-^^29^ People, especially working-age men, may be stimulated to improve their health status and quality of life if they recognize that many will live past 80 years, if they improve their health habits, and if they have a positive perception of themselves as they are.^9^^,^^30^
